# Enterovirus 71 induces degradation of TRIM38, a potential E3 ubiquitin ligase

**DOI:** 10.1186/1743-422X-8-61

**Published:** 2011-02-10

**Authors:** Xinlei Liu, Xiaobo Lei, Zhuo Zhou, Zhenmin Sun, Qinghua Xue, Jianwei Wang, Tao Hung

**Affiliations:** 1 State Key Laboratory of Molecular Virology and Genetic Engineering, Institute of Pathogen Biology, Peking Union Medical College & Chinese Academy of Medical Sciences, Beijing 100730, P.R. China

## Abstract

**Background:**

The tripartite motif (TRIM) proteins are a family of more than 70 members in human. However, only a few of them have been well studied. The TRIM proteins contain the conserved RING, B-box, coiled-coil, and SPRY domains, most of which are involved in protein ubiquitination. TRIM38 is a member of the TRIM protein family, which we studied in more detail here as its functions are largely unknown.

**Results:**

Our study shows that, similar to other TRIM family members, TRIM38 is localized in the cytoplasm. TRIM38 increases ubiquitination of other cellular proteins and catalyzes self-ubiquitination. TRIM38 also promotes K63- and K48-linked ubiquitination of cellular proteins. An intact RING domain is important for the functions of TRIM38. In addition, enterovirus 71 infection induces TRIM38 degradation.

**Conclusions:**

Our observations demonstrate that TRIM38 has E3 ubiquitin ligase activity and can be degraded during virus infection. These findings may provide insight into innate immune signaling pathways.

## Background

The tripartite motif (TRIM) proteins are involved in various cellular processes such as cell proliferation, differentiation, apoptosis, and antiviral defense [1-5]. Most members of the TRIM family contain the conserved RING domain, one or two B-box domains and coiled-coil domain (CCD) [6]. The RING domain is a specialized zinc finger of 40-60 residues in the N-terminus of most TRIM proteins. Previous studies have demonstrated that the RING finger domain of many TRIM proteins, including that of TRIM5α, TRIM22, and TRIM25, possesses an E3 ubiquitin ligase activity, which can catalyze ubiquitin to cellular proteins or viral proteins [7-10]. The E3 ubiquitin ligase activity of some TRIM proteins plays an important role in regulating innate immune signaling as well as restricting HIV replication [11-13].

Ubiquitin is a highly conserved protein consisting of 76 amino acids. It is involved in multiple cellular processes [14]. For example, ubiquitin protein conjugation represents a novel post-transcriptional modification. Furthermore, although ubiquitin contains seven lysine residues, all of which can be used to assemble various linkage polyubiquitin chains, only the functions of Lys48 (K48) and Lys63 (K63) linkage polyubiquitin chains have been well characterized. K63-linked polyubiquitin chains have been shown to be involved in stress response, protein kinase activation, protein-protein interactions and assembly of signaling complexes [15,16]; whereas K48-linked polyubiquitin chains are tagged to substrates, which are recognized by the 26S proteasome for degradation [17]. The specificity to the ubiquitin conjugation system is provided by E3 ligases through direct interaction with substrates. Some such E3 ligases are RING finger proteins [7,8].

In recent years, outbreaks of hand, foot, and mouth disease (HFMD) have occurred frequently in many countries with high mortalities, in particular in the Asia-Pacific area, and have become a great challenge to public health [18-20]. Enterovirus 71 (EV71), a single-stranded, positive-sense RNA virus belonging to the *Picornviridae*, is one of the major causative agents for HFMD [21]. Although HFMD is usually a mild disease, EV71 infection can be associated with severe neurological complications, including encephalitis, aseptic meningitis, and fatal pulmonary edema in infants and young children [19,22,23]. Thus, EV71 has become the most important neurotropic enterovirus after the eradication of poliomyelitis [23]. Effective antiviral drugs and vaccines against EV71 are currently still unavailable. To date, little is known about the mechanism of EV71 pathogenesis. It is believed that host factors are involved in the pathogenesis of EV71 infections which may affect host functions, such as translation and polyadenylation by degradation of eIF5B and CstF-64 [24,25]. EV71 can also strongly block the interferon response, an important innate immunity mechanism against virus infections [26].

An increasing number of TRIM proteins have been identified and have been found to be involved in processes associated with innate immunity [27]. TRIM38 is a member of the TRIM protein family with a RING finger domain. However, its cellular functions have not been well studied. In this report, we demonstrate that TRIM38 is a potential RING finger E3 ubiquitin ligase. We also show that EV71 infection induces degradation of TRIM38 in the cells, suggesting that TRIM38 may play a role in viral infections.

## Results

### Characterization of TRIM38

**Figure 1:**
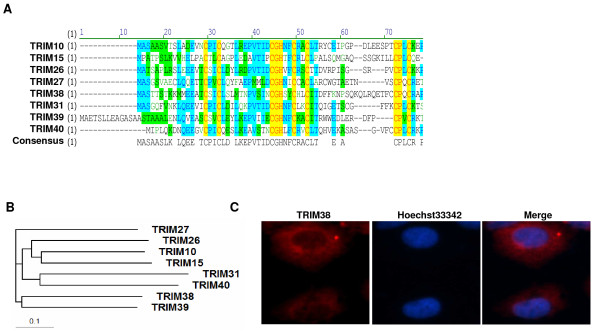
**Preliminary characterization of TRIM38. (A) Alignment of N-terminal domain TRIM proteins amino acid sequences by Vector NTI advance 9**. (B) Evolutionary tree of TRIM family members encoded by genes on human chromosome 6p21. Amino acid sequences of TRIM family members were aligned using Clustal W. The alignment was then used to build trees in MEGA4. The scale bar represents evolutionary distance in amino acid residues. (C) TRIM38 was visualized via labeling with polyclonal rabbit anti-TRIM38, followed by labeling with anti-rabbit IgG/DyLight 594 conjugated secondary antibodies. Representative confocal images are shown.

Analysis by Map Viewer software on the NCBI website showed that the TRIM38 gene is located on human chromosome 6p21.3, and clustered with other TRIM genes including TRIM10, TRIM15, TRIM26, TRIM27, TRIM31, TRIM39, and TRIM40 (data not shown). The RING-domain, which is located at the N-terminus of TRIMs, is critical to the activity of most E3 ligase [7,8]. To characterize the similarity between TRIM38 and other TRIMs, amino acid sequences of the TRIM proteins with their N-terminal domain were aligned using Vector NTI Advance 9. The results show that these TRIMs all possess a RING-consensus sequence (Figure 1A). The RING domain is a type of zinc finger which contains a Cys3HisCys4 (yellow area, Figure 1A) amino acid motif that binds two zinc cations [28]. Phylogenetic analysis suggests that TRIM38 is closely related to TRIM39 and TRIM27. TRIM38 and TRIM39 share 37% amino acid identity, and TRIM38 shares 34% amino acid identity with TRIM27 throughout the protein sequence (Figure 1B).

We then examined the subcellular localization of endogenous TRIM38 in A549 cell lines, which have a relatively high expression of TRIM38, by first incubating A549 cells with polyclonal rabbit anti-TRIM38 antibody, followed by labeling with anti-rabbit IgG/DyLight 594 conjugated secondary antibodies. As shown in Figure 1C, TRIM38 was detected mainly in the cytoplasm, but hardly in the nucleus. These results indicated that the subcellular localization of TRIM38 is similar to other TRIM proteins such as TRIM27 and TRIM39 [29,30].

### TRIM38 functions as an E3 enzyme and self-ubiquitinates *ex vivo*

**Figure 2:**
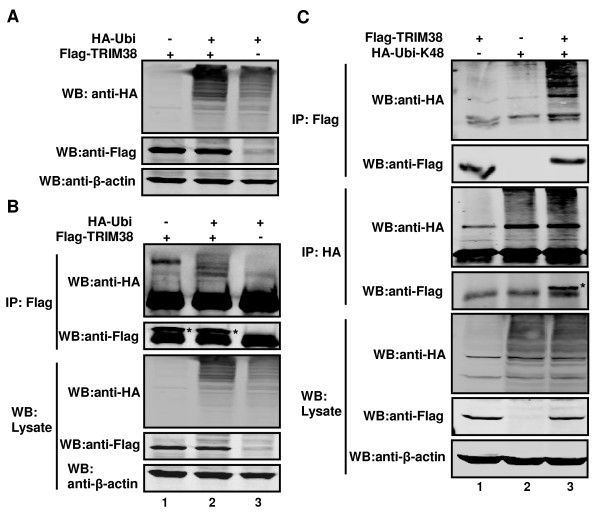
**TRIM38 functions as an E3 enzyme and ubiquitinates itself *ex vivo***. (A) TRIM38 catalyzes the ubiquitination of cellular proteins. 293T cells were transfected with plasmids expressing Flag-TRIM38 (lanes 1 and 2), pRK5-HA-Ubiquitin (lanes 2 and 3). Whole cell lysates were subjected to Western blot analysis with antibodies against HA, Flag and β-actin. (B) TRIM38 is self-ubiquitinated. Similar to (A), except that cell lysates were immunoprecipitated with anti-Flag. Immunoprecipitates and aliquots of cell lysates were subjected to Western blot analysis with antibodies against HA, Flag, and β-actin. (C) TRIM38 mediates itself K48-ubiquitination. 293T cells were transfected with plasmids expressing Flag-TRIM38 (lanes 1 and 3), pRK5-HA-ubiquitin-K48 (lanes 2 and 3). Cell lysates were immunoprecipitated with anti-Flag or anti-HA. Immunoprecipitates and aliquots of cell lysates were subjected to Western blot analysis with antibodies against HA, Flag and β-actin.

Many RING finger domain-containing proteins have been shown to bind ubiquitin enzymes or their substrates, hence functioning as E3 ligases [7-9]. As TRIM38 has a RING-finger domain which is located between Cys-16 and Cys-62 at its N-terminal region, we hypothesized that TRIM38 functioned as an E3 ubiquitin ligase. To test this, 293T cells were transiently transfected with Flag-tagged-TRIM38 and pRK5-HA-ubiquitin-WT polypeptide (HA-ubi). Subsequently, whole cell extracts were analyzed by Western blot using anti-HA polyclonal antibodies in order to detect HA-tagged ubiquitin conjugated proteins. The expression of Flag-tagged TRIM38 was probed with anti-Flag monoclonal antibody. As shown in Figure 2A, ubiquitin conjugates were produced in cells transfected with HA-ubi alone, reflecting ubiquitination by endogenous ubiquitin ligase. The level of conjugates was markedly increased in the presence of Flag-tagged-TRIM38 (Figure 2A). Our data demonstrated that TRIM38 could catalyze the ubiquitination of cellular proteins.

Recently, several groups reported that E3 ubiquitin ligases are ubiquitinated by themselves [7,9]. Thus, we evaluated whether TRIM38 can be self-ubiquitinated. For this purpose, HA-ubi vector was co-expressed with Flag-tagged-TRIM38, and co-immunoprecipitation assays were performed. Immunoblot analysis revealed that Flag-tagged-TRIM38 was ubiquitinated when co-expressed with HA-Ubi (Figure 2B). These results suggest that TRIM38 can be ubiquitinated by itself.

Substrates that are recognized by the 26S proteasome are tagged by K48-linked polyubiquitin chains [17]. As mentioned before, TRIM38 could be ubiquitinated. Thus, we tested whether TRIM38-mediated cellular proteins degradation was promoted by K48-linked ubiquitination by co-transfecting Flag-TRIM38 with pRK5-HA-ubiquitin-K48 (HA-Ubi-K48) into 293T cells, and ubiquitinated proteins were immunoprecipitated using anti-Flag or anti-HA antibodies. Immunoprecipitated poly-ubiquitinated TRIM38 was detected using the anti-Flag and anti-HA antibodies. Western blot analysis revealed that TRIM38 mediated its own K48-ubiquitination (Figure 2C) when co-expressed with HA-Ubi-K48.

### RING domain is important for ubiquitin activity of TRIM38

**Figure 3:**
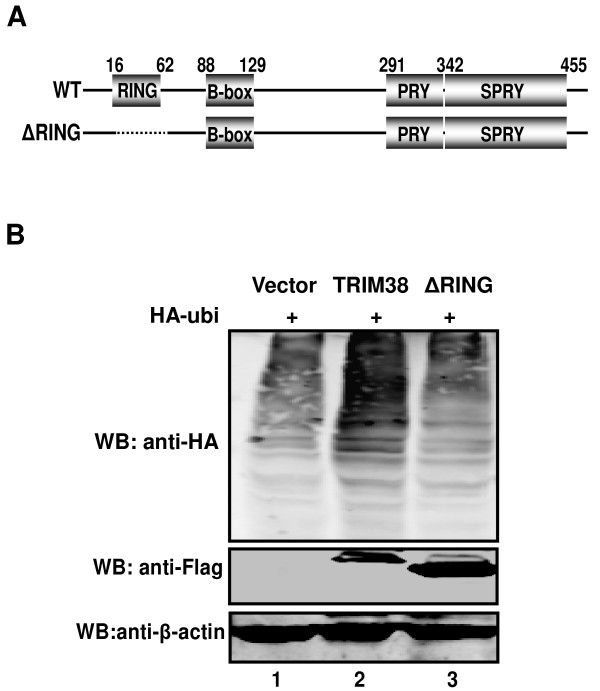
**RING domain is critical to ubiquitin activity of TRIM38**. (A) Schematic representation of TRIM38 and TRIM38-deletion mutants. (B) 293T cells transfected with plasmids expressing Flag-TRIM38 (lanes 2), Flag-ΔRING (lanes 3), HA-Ubiquitin (lanes1, 2 and 3). Whole cell lysates were subjected to Western blot analysis with antibodies against HA, Flag and β-actin.

Many RING-finger proteins have been identified as E3 ubiquitin ligases [7-9]. This E3 activity of RING-finger proteins can be abolished by deletion of the conserved RING domain. To evaluate the possible roles of the RING domain in the ubiquitin-conjugating activity of TRIM38, we constructed a TRIM38 mutant in which the RING domain was deleted, Flag-TRIM38-ΔRING (ΔRING, deletion from residues 16 to 62) (Figure 3A). HA-ubi vector was co-expressed with Flag-TRIM38 or Flag-TRIM38-ΔRING. Cells were collected and whole-cell extracts were analyzed by Western blot. As expected, no ubiquitination activity was detected with TRIM38-ΔRING compared to the wild type TRIM38 (Figure 3B). The results strongly suggest that the RING-finger domain is required for TRIM38 to catalyze ubiquitin-conjugation of cellular proteins.

### TRIM38 catalyzes K63-linked ubiquitin to cellular proteins

**Figure 4:**
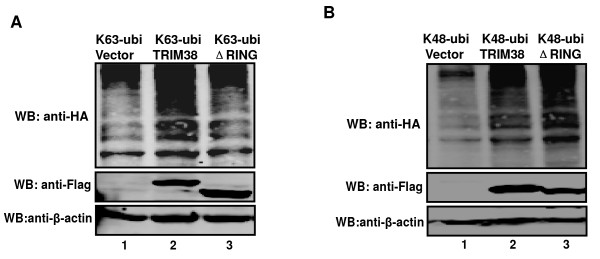
**TRIM38 catalyzes K63-linked ubiquitin to cellular proteins**. (A) 293T cells were transfected with plasmid expressing Flag-TRIM38 (lanes 2), or Flag-ΔRING (lanes 3) and pRK5-HA-ubiquitin-K63-linked-Ubiquitin (lanes 1, 2, and 3). Whole cell lysates were subjected to Western blot analysis with antibodies against HA, Flag and β-actin. (B) Similar to (A), except that pRK5-HA-ubiquitin-K48-linked-Ubiquitin was used instead of pRK5-HA-ubiquitin-K63-linked-Ubiquitin.

Ubiquitin contains seven lysine residues, all of which can be used to assemble various linkage specific polyubiquitin chains. However, only K48 and K63 polyubiquitin linkages have been well determined in cells. The K63-linked chains function in cell signaling pathways and the K48-linked chains are used for proteasomal degradation [15-17]. To test whether TRIM38 could influence both or one of the two types of polyubiquitin linkages, we co-expressed Flag-TRIM38 or Flag-TRIM38-ΔRING with K63-linked ubiquitin and K48-linked ubiquitin in 293T cells. As shown in Figure 4, TRIM38 facilitated synthesis of both K63- and K48- linked polyubiquitin chains. But the activity of TRIM38 facilitating synthesis of K63-linked polyubiquitin chains could be abolished by deletion of the conserved RING domain (Figure 4A), however, the K48-linked polyubiquitination is not affected if the RING domain of TRIM38 is deleted (Figure 4B). Our data demonstrated that the RING domain of TRIM38 is needed to catalyze the K63-linked ubiquitin to cellular proteins.

### EV71 infection promotes degradation of TRIM38

**Figure 5:**
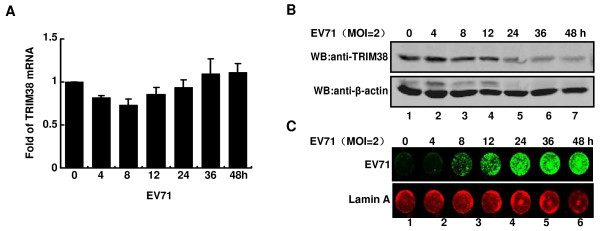
**Enterovirus 71 infection promotes degradation of TRIM38**. (A) Detection of TRIM38 mRNA. A549 cells were mock-infected or infected with EV71 at 2 MOIs. At indicated time points, total RNA was extracted from cells. TRIM38 transcription level was analyzed by quantitative real-time PCR using SYBR-Green. GAPDH gene primer sets were used as an internal control. (B) Detection of TRIM38 protein. At 4, 8, 12, 24, 36 and 48 h post-infection of EV71, A549 cells were harvested and whole cell lysates were prepared for Western blot analysis with antibodies against TRIM38 and β-actin. (C) Detection of EV71 proteins. At 4, 8, 12, 24, 36 and 48 h post-infection of EV71, cells were subjected to In-cell Western blot analysis. Shown are representative images with the green color depicting EV71 proteins and the red color depicting lamin A, which was used as an internal control.

TRIM family proteins are associated with virus-host interaction and antiviral activities, especially in IFN responses [10,31]. Expression of several TRIM proteins was shown to be altered after treatment with IFN. TRIM38 expression was up-regulated by type I IFN in peripheral blood lymphocytes and monocyte derived macrophages [32]. To examine whether TRIM38 expression is affected upon virus infection, we analyzed the TRIM38 expression level in response to EV71 infection. For A549 cells infected with EV71 (MOI = 2), qRT-PCR analysis was performed to determine the mRNA level of TRIM38, using GAPDH as an internal control. As indicated in Figure 5A, EV71 did not influence the RNA level of TRIM38. In parallel to the qRT-PCR analysis, protein expression levels of TRIM38 were examined by Western blot. We found that EV71 infection reduced the amount of TRIM38 protein (Figure 5B). Similarly, we found that Sendai viral infection led to a decrease in TRIM38 expression (data not shown). These observations were associated with the replication of EV71, as levels of EV71 proteins increased with time in A549 cells as seen by in-cell Western blot analysis (Figure 5C). Taken together, these findings indicate that EV71 infection reduces the TRIM38 expression at the protein level rather than at RNA level and that TRIM38 may play a role in virus infection.

## Discussion

TRIM38 has been shown to be capable of activating NF-κB signaling pathways [33]. In this study, we performed *ex vivo *ubiquitylation assays to evaluate if TRIM38 functions as an E3 ligase, and found that TRIM38 can increase the ubiquitination of cellular proteins and be self-ubiquitinated in the cells. Further, we demonstrated that the ability of TRIM38 to increase the level of ubiquitination depends on its RING finger domain. In addition, we demonstrated that TRIM38 is located in the cytoplasm, and its expression is reduced by EV71 infection. These results support the notion that TRIM38 is a potential RING finger E3 ubiquitin ligase and it may participate in virus-host interactions.

Many TRIM proteins have an E3 ligase activity [34]. For example, TRIM25 could markedly increase the ubiquitination level of exogenous RIG-I (Retinoic acid-inducible gene I), and potentiate the production of RIG-I mediated interferon-β to elicit host antiviral response [8,35]. TRIM22 functions as an E3 ubiquitin ligase in a RING finger dependent manner [7], and is required to mediate antiviral activity against encephalomyocardites virus and HIV [10,31]. TRIM5α was also shown to be crucial for anti-HIV function [36,37], and TRIM19/promyelocytic leukemia (PML) has been identified as a cellular factor against viral infection [38]. Here we show that N-terminal RING domain of TRIM38 is necessary for its potential E3 ligase activity. This result is consistent with that of other E3 ubiquitin ligases of the TRIM family proteins. However, the substrate(s) of TRIM38 other than itself remains unknown.

Ubiquitin conjugation represents a novel pattern of posttranscriptional modification and is involved in regulation of different cellular processes in addition to proteolytic degradation. It has been demonstrated that K63-linked polyubiquitination chains are involved in TAK1 (Transforming growth factor β-activated kinase 1) kinase complex activation and IKK (the IκB kinase) phosphorylation [39,40]. TRAF6 (Tumor necrosis factor receptor associated factor 6) is also an E3 ubiquitin ligase and specifically catalyzes K63-linked polyubiquitin chains on target proteins, including NEMO (NF-kappa-B essential modulator) and TRAF6 itself to activate the type I interferon signaling pathways [41]. Based on our observations in this study, we can speculate that TRIM38 may facilitate the assembly of a K63-linked polyubiquitin chain in a RING domain dependent manner and thus participate in regulating innate immune signaling pathways.

E3 ligase has been shown to interact with virus proteins and is involved in antiviral activity during viral infection. For example, TRIM22 was shown to interact with the 3C protease of encephalomyocarditis virus and mediate its ubiquitination against picornaviruses [10]. Our observations demonstrate that the expression of TRIM38 protein is regulated after viral infection. Therefore, TRIM38 may play a role in the virus infection.

In conclusion, TRIM38 act as a potential E3 ubiquitin ligase. We propose that TRIM38 catalyzes its substrates through ubiquitylation in the cytoplasm, thus regulating signaling pathways or interacting with virus proteins to interfere with virus replication. The precise role and mechanism of TRIM38 in ubiquitylation and virus infection need to be further addressed.

## Methods

### Cell lines and virus

The human lung carcinoma cell line A549 and human embryo kidney cells 293T were maintained in Dulbecco's modified Eagle's medium (DMEM; Invitrogen, Carlsbad, CA) supplemented with 10% fetal bovine serum (FBS) (HyClone, Logan, UT), 100 U/ml penicillin, and 100 μg/ml streptomycin at 37°C in a 5% CO_2 _humidified atmosphere. The EV71 strain used in this study (Shenzhen 98), kindly provided by Dr. Qi Jin, was described previously [27]. Virus infection was carried out as follows. Briefly, A549 cells were infected with EV71 at the indicated multiplicity of infection (MOI). Viruses were washed away after 2 h, and cells were then cultured with fresh medium supplemented with 2% FBS. At the indicated times post infection, cell lysates were harvested and were subjected to Western blot analysis.

### Plasmids

The TRIM38 expressing plasmid pCMV6-Entry-TRIM38 was purchased from Origene Technologies (Rockville, MD). The Flag-tagged-TRIM38, Flag-TRIM38-ΔRING, deletions were constructed by using a site-directed mutagenesis kit (Stratagene, La Jolla, CA) using the pCMV6-Entry-TRIM38 vector following the protocols provided by the manufacturer. The ubiquitin expression plasmids pRK5-HA-ubiquitin-WT, pRK5-HA-ubiquitin-K63, pRK5-HA-ubiquitin-K48 were made available through Addgene http://www.addgene.org.

### Computational analysis of TRIM family members

For phylogenetic analysis, amino acid sequences of TRIM family members encoded by genes on human chromosome 6p21.3 and 6p22 were aligned using Vector NTI Advance 9 (InforMax) and Clustal W [42]. The alignment was then used to build phylogenetic trees in MEGA 4 [43]. The following amino acid sequences of human TRIM family members were included in the phylogenetic analysis, including TRIM10 (GenBank accession number: NP_006769.2), TRIM15 (NP_150232.2), TRIM26 (NP_003440.1), TRIM27 (NP_006501.1), TRIM31 (NP_008959.3), TRIM38 (NP_006346.1), TRIM39 (NP_067076.2), and TRIM40 (NP_619645.1).

### Confocal immunofluorescence microscopy

A549 cells were fixed with 4% paraformaldehyde for 20 min, permeabilized with 0.1% Triton X-100 for 15 min at room temperature, and then blocked with 5% BSA for 1 h. The cells were then incubated with anti-TRIM38 polyclonal antibody (Sigma-Aldrich, St. Louis, MO) overnight at 4°C. After cells were washed with PBS, they were incubated with Alexa Fluor-594 goat anti-rabbit secondary antibody (Invitrogen, Carlsbad, CA) and washed three times with PBS. Confocal microscopy was performed using a Leica TCS SP5 laser-scanning microscope (Leica Microsystems).

### Western blot

Cells were pelleted by centrifugation and lyzed in buffer containing 150 mM NaCl, 25 mM Tris (pH 7.4), 1% NP-40, 0.25% sodium deoxycholate, and 1 mM EDTA as well as a proteinase inhibitor cocktail (Roche, Indianapolis, IN). Aliquots of cell lysates were resolved on 10% SDS PAGE and transferred to a nitrocellulose membrane (Pall, Port Washington, NY). The membranes were blocked with 5% nonfat dry milk and then incubated with anti-Flag (Sigma-Aldrich, St. Louis, MO), anti-HA (Sigma) and anti-β-actin (Sigma) primary antibodies at 4°C overnight. This was followed by incubation with the corresponding IRD Fluor 800-labeled IgG or IRD Fluor 680-labeled IgG secondary antibody (Li-Cor Inc., Lincoln, NE). After the membranes were washed with 0.1% Tween20 in PBS, they were scanned by using an Odyssey Infrared Imaging System (Li-Cor) at a wavelength of 700 to 800 nm and analyzed with Odyssey software. The molecular sizes of the developed proteins were determined by comparison with pre-stained protein markers (Fermentas, Maryland, CA).

### Immunoprecipitation

Cells were collected at 48 h after transfection and lyzed with 25 mM Tris-HCl buffer (pH 7.4) containing 150 mM NaCl, 1% NP-40, 0.25% sodium deoxycholate, 1 mM EDTA and proteinase inhibitor cocktail (Roche, Indianapolis, IN). Whole-cell lysates of cells were used for immunoprecipitation with monoclonal antibodies against Flag or HA (Sigma, St. Louis, MO) in the presence of protein A/G agarose beads (Santa Cruz Biotechnology, Santa Cruz, CA). Generally, 1-2 μg of commercial antibody was added to 500 μl of cell lysates, which was incubated at 4°C overnight. Immunocomplexes captured on the affinity gel or protein A/G agarose beads were extensively washed with lysis buffer and eluted with SDS loading buffer by boiling for 5 min. Then the samples were subjected to SDS-PAGE and Western blot analysis.

### In-cell Western blot

A549 cells were plated on 96-well plates at a density of 10^4 ^cells per well. The next day, cells were infected with EV71 at MOI of 2. At 4, 8, 12, 24, 36 and 48 h post infection, cells were fixed with 4% paraformaldehyde for 20 min, and permeabilized with 0.5% Triton X-100 for 10 min at room temperature. After cells were washed with PBS, they were incubated with mouse antibody against whole EV71 virion (Chemicon, Temecula, CA; this antibody can only used for immunofluorescence or In-cell Western blot) and rabbit anti-lamin A (Sigma-Aldrich, St. Louis, MO; used as internal reference) overnight at 4°C. Then, cells were washed with 0.1% Tween-20 in PBS and incubated with goat anti-mouse 800 (1:500) and goat anti-rabbit 680 (1:500) (Li-Cor). Cells were scanned by using an Odyssey Infrared Imaging System (Li-Cor) at a wavelength of 700 to 800 nm and analyzed with Odyssey software.

### Real-time quantitative PCR

A549 cells were mock-infected or infected by EV71 at an MOI of 2. At different time points after infection, total RNA was isolated from cells using TRIzol reagent (Invitrogen). cDNA was generated from total RNA using a Superscript cDNA synthesis kit (Invitrogen) according to the manufacturer's instructions. Real-time PCR was performed using SYBR^® ^PrimeScript™ RT-PCR Kit II (Takara) following the manufacturer's instructions. The primer sequences used for qRT-PCR were as follows: TRIM38, 5'-TTTGAGCAGGAGTTGGGC-3'(forward), 5'-GCTTGGAAACATTGCACATA-3'(reverse); GAPDH, 5'-GCTTGGAAACATTGCACATA-3'(forward), 5'-AGGGGCCATCCACAGTCTTC-3'(reverse).

## Competing interests

The authors declare that they have no competing interests.

## Authors' contributions

XL, XL, ZZ, ZS, and QX carried out the experiments and wrote the manuscript. XL, JW and TH designed the research and reviewed the drafts. All authors read and approved the final manuscript.
